# Host-dependent symbiotic efficiency of *Rhizobium leguminosarum* bv. *trifolii* strains isolated from nodules of *Trifolium rubens*

**DOI:** 10.1007/s10482-017-0922-7

**Published:** 2017-08-08

**Authors:** Monika Marek-Kozaczuk, Sylwia Wdowiak-Wróbel, Michał Kalita, Mykhaylo Chernetskyy, Kamil Deryło, Marek Tchórzewski, Anna Skorupska

**Affiliations:** 1 Department of Genetics and Microbiology, Maria Curie-Skłodowska University, Akademicka 19, 20-033 Lublin, Poland; 2 The Botanic Garden of Maria Curie-Skłodowska University, Sławinkowska 3, 20-810 Lublin, Poland; 3 Department of Molecular Biology, Maria Curie-Skłodowska University, Akademicka 19, 20-033 Lublin, Poland

**Keywords:** *Trifolium rubens*, Rhizobia, Symbiosis, MLSA

## Abstract

*Trifolium rubens* L., commonly known as the red feather clover, is capable of symbiotic interactions with rhizobia. Up to now, no specific symbionts of *T. rubens* and their symbiotic compatibility with *Trifolium* spp. have been described. We characterized the genomic diversity of *T. rubens* symbionts by analyses of plasmid profiles and BOX–PCR. The phylogeny of *T. rubens* isolates was inferred based on the nucleotide sequences of 16S rRNA and two core genes (*atpD, recA*). The *nodC* phylogeny allowed classification of rhizobia nodulating *T. rubens* as *Rhizobium leguminosarum* symbiovar *trifolii* (*Rlt*). The symbiotic efficiency of the *Rlt* isolates was determined on four clover species: *T. rubens, T. pratense*, *T. repens* and *T. resupinatum.* We determined that *Rlt* strains formed mostly inefficient symbiosis with their native host plant *T. rubens* and weakly effective (sub-optimal) symbiosis with *T. repens* and *T. pratense*. The same *Rlt* strains were fully compatible in the symbiosis with *T. resupinatum*. *T. rubens* did not exhibit strict selectivity in regard to the symbionts and rhizobia closely related to *Rhizobium grahamii, Rhizobium galegae* and *Agrobacterium radiobacter*, which did not nodulate *Trifolium* spp., were found amongst *T. rubens* nodule isolates.

**Electronic supplementary material:**

The online version of this article (doi:10.1007/s10482-017-0922-7) contains supplementary material, which is available to authorized users.

## Introduction

In their symbiotic association with legume plants, rhizobia have the potential to fix nitrogen in amounts sufficient to reduce the dependence of plants on nitrogen fertilizers (Herridge 2008). They are distributed worldwide in many types of soil where they can be found as free-living organisms or symbionts of leguminous plants. *Rhizobium*-legume symbiosis plays a critical role in sustainable agriculture, because it reduces the need for nitrogen fertilizer while ensuring efficient protein-rich production. Rhizobia attach to the root hairs of plants, invade plant tissues, and colonize the cells, forming nodules where they differentiate into nitrogen-fixing bacteroids. The *Rhizobium*-legume symbiosis is specific and depends on the exchange of signal molecules, such as flavonoids, secreted by plants, which induce expression of bacterial nodulation (*nod*) genes via interaction with the NodD regulatory protein (Perret et al. 2000; Jones et al. 2007; Oldroyd and Downie 2008; Wang et al. 2012). The Nod proteins synthesize lipochitin oligosaccharides (Nod factors) recognized by host plant receptors and, in response, tubular structures called infection threads are formed where bacteria proliferate and are released into plant nodule cells forming symbiosomes. In these structures, bacteria differentiate into nitrogen-fixing bacteroids and turn N_2_ into ammonia, which is assimilated by the legume host (Heidstra and Bisseling 1996; Gage and Margolin 2000). In the indeterminate nodules formed by galegoid plants, five developmental zones are distinguished: the apical meristem functioning during nodule development (I), the invasion zone (II) into which infection threads release rhizobia, the interzone (II–III), the nitrogen-fixing zone (III), the senescence zone (IV), and the saprophytic zone (V) in older nodules (Vasse et al. 1990; Timmers et al. 2000). In the saprophytic zone, bacteroids degenerate and non-nitrogen fixing, undifferentiated rhizobia are released from infection threads, which increase the rhizobial population in the rhizosphere after nodule senescence (Timmers et al. 2000; Wielbo et al. 2010a, b). Thus, the nitrogen-fixing nodule is an organ where reciprocal benefits for both partners occur in different regions of the nodules. Although the legume-*Rhizobium* symbiosis is beneficial to the host, the nitrogen-fixation efficiency significantly varies between different plant-*Rhizobium* interactions and the molecular mechanisms of strain-specific nitrogen fixation are largely unknown (Schumpp and Deakin 2010; Wang et al. 2012). Plants play a significant role in the control of later stages of symbiosis, such as bacteroid differentiation inside nodules of some galegoid (IRLC) plants (*Medicago, Trifolium, Vicia, Pisum, Astragalus*) and endoreduplication of bacterial genomes forming bacteroids (Mergaert et al. 2006; Haag et al. 2013). At this stage, bacteroids exhibit decreased cytoplasmic membrane integrity and undergo terminal differentiation in relation to their free-living form while maintaining the metabolic activity required for nitrogen fixation and nutrient exchange with the host plant. Bacteroid differentiation studied in *Medicago truncatula* is mediated by a large family of legume nodule-specific cysteine-rich (NCR) peptides transported to symbiosomes, which have antimicrobial activity in vitro and have a critical role in bacteroid development and persistence in vivo (Haag et al. 2011; Van De Velde et al. 2010; Kondorosi et al. 2013).

The genome of *Rhizobium leguminosarum* is large and complex, consisting of a chromosome and a variable number of large plasmids (Young et al. 2006; Mazur et al. 2011; Kumar et al. 2015). Symbiotic functions are encoded by genes located in symbiotic plasmids (pSym) (Perret et al. 2000; Young et al. 2006; Mazur et al. 2011, 2013). The plasmids constitute a pool of accessory genetic information and contribute to the plasticity and dynamic state of the genome commonly observed among members of the *Rhizobiaceae* family (Palacios and Newton 2005). The host range of *R. leguminosarum* (*Rl*) species varies; *R. leguminosarum* bv. *viciae* (*Rlv*) is able to induce efficient symbiosis with legumes belonging to the genera *Pisum, Vicia, Lathyrus,* and *Lens* forming several species and biovars (symbiovars) (Laguerre et al. 2003; Alvarez-Martinez et al. 2009; Ramirez-Bahena et al. 2009; Rogel et al. 2011; Rashid et al. 2015). The development of effective symbiotic associations of *Rlv* with the large group of legume plants indicates that both partners contain compatible determinants for successful nodulation and N_2_ fixation. In contrast to *Rlv, R. leguminosarum* bv. *trifolii* (*Rlt*) symbiotic host range is confined to the clover genus (*Trifolium* spp.) (Ramirez-Bahena et al. 2008; Reeve et al. 2010a, b; Kumar et al. 2015).

The clover genus *Trifolium* L. is a member of a large clade of legumes lacking one copy of the chloroplast—inverted repeat (IRLC) and is one of the largest genera in the family consisting of approximately 255 herbaceous species (Watson and Sayed-Ahmed 2000; Ellison et al. 2006). These are commonly cultivated forage and green manure crops, which grow in a great range of soils with the highest species diversity in the temperate climate (Ellison et al. 2006). *Rlt* - *Trifolium* sp. symbiotic associations are mostly effective; however, host-dependent ineffective nodulation is evident amongst *Rlt* strains that nodulate clover species. Some *Rlt* strains form effective nodules on *Trifolium repens*, *Trifolium pratense*, and *Trifolium subterraneum*, whereas other strains are ineffective on *Trifolium subterraneum* (Tesfaye and Holl 1999). There are *Rlt* strains effective on *T. subterraneum,* but ineffective or partially effective on *T. repens* and *T. pretense* (Elliot et al. 1998). In another case, *Rlt* strains are able to form effective nodules on *Trifolium ambiguum* (Caucasian clover) but ineffective ones on *T. repens, T. pratense* and vice versa (Beauregard et al. 2004; Miller et al. 2007). Melino et al. (2012) reported sub-optimal or ineffective symbiotic association between an *Rlt* strain and *T. subterraneum*, *T. purpureum*, and *Trifolium polymorphum*. These reports show that rhizobial strains commonly found in agricultural soils are frequently poorly compatible with commercially grown clover species but little is known of the cause of their ineffectivity.

One of the clover species is *Trifolium rubens* L., commonly known as the red feather. This is a perennial, ornamental, xerothermic species of clover growing in normal soil in Central and South Europe. In Poland, *T. rubens* belongs to rare species whose occurrence has significantly decreased (86%) in the recent years (Michalik 2009). Like other clover species, it is capable of symbiotic association with rhizobia; however, to the best our knowledge, no specific symbionts of *T. rubens* species and their symbiotic efficiency have been described.

The primary goal of the study was to investigate the symbiotic compatibility of *T. rubens* nodule isolates with four agronomically important *Trifolium* spp. Moreover, genomic diversity and phylogeny of *T. rubens* symbionts were established. The symbiotic phylogeny inferred based on the *nodC* of *R. leguminosarum* nodulating *T. rubens* classified the isolates as belonging to the biovar *trifolii.* In clover plant tests, *T. rubens* isolates formed ineffective or weakly effective symbiosis with their native host. Their symbiotic efficiencies with other species i.e. *T. repens, T. pratense* and *Trifolium resupinatum* were also varied. *T. rubens* seems to not exhibit strict selectivity in regard to the symbionts and other rhizobia closely related to *Rhizobium grahamii, Rhizobium galegae* and *Agrobacterium radiobacter*, which were not able to nodulate four studied *Trifolium* spp., were found amongst *R. leguminosarum* bv*. trifolii* nodule isolates.

## Materials and methods

### Bacterial strains and growth conditions

Rhizobia were isolated from root nodules of *T. rubens* L. grown in meadows in the region of Lublin, Poland. The bacteria were isolated from surface-sterilized root nodules as follows: nodules were intensively washed in water and sterile distilled water and then nodules were sterilized by 3 min in 3% sodium chlorite and washed repeatedly in sterile distilled water. After surface sterilization, the nodules were crushed and their contents were plated on solid 79CA medium (Vincent 1970). The bacteria isolated from nodules were purified by repeatedly streaking of single colonies and pure cultures were used in further experiments.

### DNA analyses

Plasmid content analyses of the nodule isolates were performed as described by Eckhardt (1978). Estimation of plasmids size was performed using Bio-Profile V11.01 (Vilber-Lourmat, France) compared with standard plasmids of *R. leguminosarum* bv. *viciae* strain 3841 (Young et al. 2006). Genomic DNA of each isolate was extracted from 5 ml of a 2-day bacterial culture in liquid TY using the method of Pitcher et al. (1989). PCR reactions were carried out using the Ready Mix Taq PCR Reaction (Sigma) according to the manufacturer’s recommendations. The primers and protocols used for amplification and sequencing the genes encoding 16S rRNA, *atpD* and *recA* (Weisburg et al. 1991; Louws et al. 1994; Gaunt et al. 2001; Martens et al. 2008) and for BOX-PCR are described in Table S1. Standard techniques were used for DNA labeling and Southern hybridization (Sambrook et al. 1989). DNA probe *nodC* for Southern hybridization was obtained by PCR amplification with *R. leguminosarum* bv. *trifolii* TA1 genomic DNA as the template and appropriate primers (Laguerre et al. 2001) (Table S1). The hybridization probe was labeled with the non-radioactive DIG DNA Labeling and Detection Kit (Roche). Automatic sequencing of PCR products was performed using the BigDye™ Terminator Cycle Sequencing Kit and an ABI Prism 3730 XL Genetic Analyzer (Applied Biosystems) according to manufacturer’s instructions. BOX-PCR genomic fingerprints were obtained as described by Louws et al. (1994). The sequences were aligned with those from GenBank using the MEGA5.05 software package (Tamura et al. 2011). The distances were calculated according to the Kimura’s 2-parameters (Kimura 1980). Phylogenetic trees were inferred using neighbor-joining method (NJ). Bootstrap analyses were calculated based on 1000 replications (Felsenstein 1985). Chromosomal 16S rRNA and the house-keeping genes *atpD* and *recA*, and symbiotic *nodC* sequences of *T. rubens* isolates determined in this study have been deposited in the GenBank under following accession numbers: KU715819-KU715834, KU714648-KU714656, KU714675-KU714689.

### Plant growth experiments

To examine the symbiotic effectiveness of *T. rubens* isolates, four clover species were used as host plants: red feather (*T. rubens* L.), red clover (*T. pratense* L. cv. Rozeta), white clover (*T. repens* cv. Lipollo), and Persian clover (*T. resupinatum* L. Lightning). The seeds were surface sterilized by 15 min in 2% sodium hypochlorite, washed in sterile distilled water, and germinated on 0.8% agar-water plates. Four-day-old clover seedlings were planted in sterile nitrogen-free slants (one per tube) and 2 days later healthy seedlings were inoculated with ~10^8^ cells of an individual strain grown in liquid 79CA, pelleted, and suspended in sterile water. Ten replicates were prepared in glass tubes for each rhizobial strain-clover species. The plants were grown for 5 weeks in a greenhouse under natural light supplemented with artificial light (14 h day/10 h night, at 24/19 °C). Then, the number of nodules and the fresh weight of shoots were examined. Symbiotic effectivity was estimated based on nodule number and the fresh shoot weight inoculated and uninoculated (growing without nitrogen) control plants.

A greenhouse experiment was carried out in plastic pots (500 cm^3^) filled with sterilized mixture of sand and vermiculite in the ratio 3:1. One day before seeding, each pot (two pots for each strain/clover species) was fertilized with 150 ml of Fahraeus N-free medium (negative control, N^−^) or the medium supplemented with 0.1% KNO_3_ (positive control, N^+^). Seeds of the four *Trifolium* spp. were surface sterilized for 15 min in 2% sodium hypochlorite, and then repeatedly washed with sterile distilled water. After sterilization, forty legume seeds per pot were seeded. After 5 days each pot was inoculated with 10 ml of rhizobia culture (~10^8^ cfu ml^−1^). Plants were watered once a week with sterilized water and maintained under greenhouse conditions (day/night temperature: 23/18 °C; 12 h light/12 h dark period; relative humidity 70–80%). Plants were harvested 40 days after planting; wet mass and nodule number of thirty plants from each of the pots were estimated. The results of plant tests were analyzed with the Student’s test at a significance level of p < 0.05 and used for statistical analysis.

### Confocal microscopy

Images of the root nodule sections were collected on a laser scanning confocal microscope LSM780 Zeiss with ZEN2010 data acquisition software using a EC Plan—Neofluar 10x/0.30 M27 objective. Sequential two-channel imaging in tile scan mode was performed with excitation light set at 488 nm from an Argon laser for SYTO9 and at 561 nm from a DPSS laser for Propidium Iodide (PI). Fluorescence emission was recorded in the range of 490–590 and 600–690 nm, respectively. Both lasers worked at 2% power to avoid photobleaching. Pinhole diameter was set to 1 AU. In situ live/dead staining of hand nodule sections was performed by incubation for 20 min in live/dead staining solution (5 μM SYTO9 and 30 μM PI in 50 mM Tris pH 7.0 buffer. Section were removed from the staining solution and mounted in deionized water for microscopy observation.

## Results

### Genomic diversity of *T. rubens* nodule isolates

**Fig. 1:**
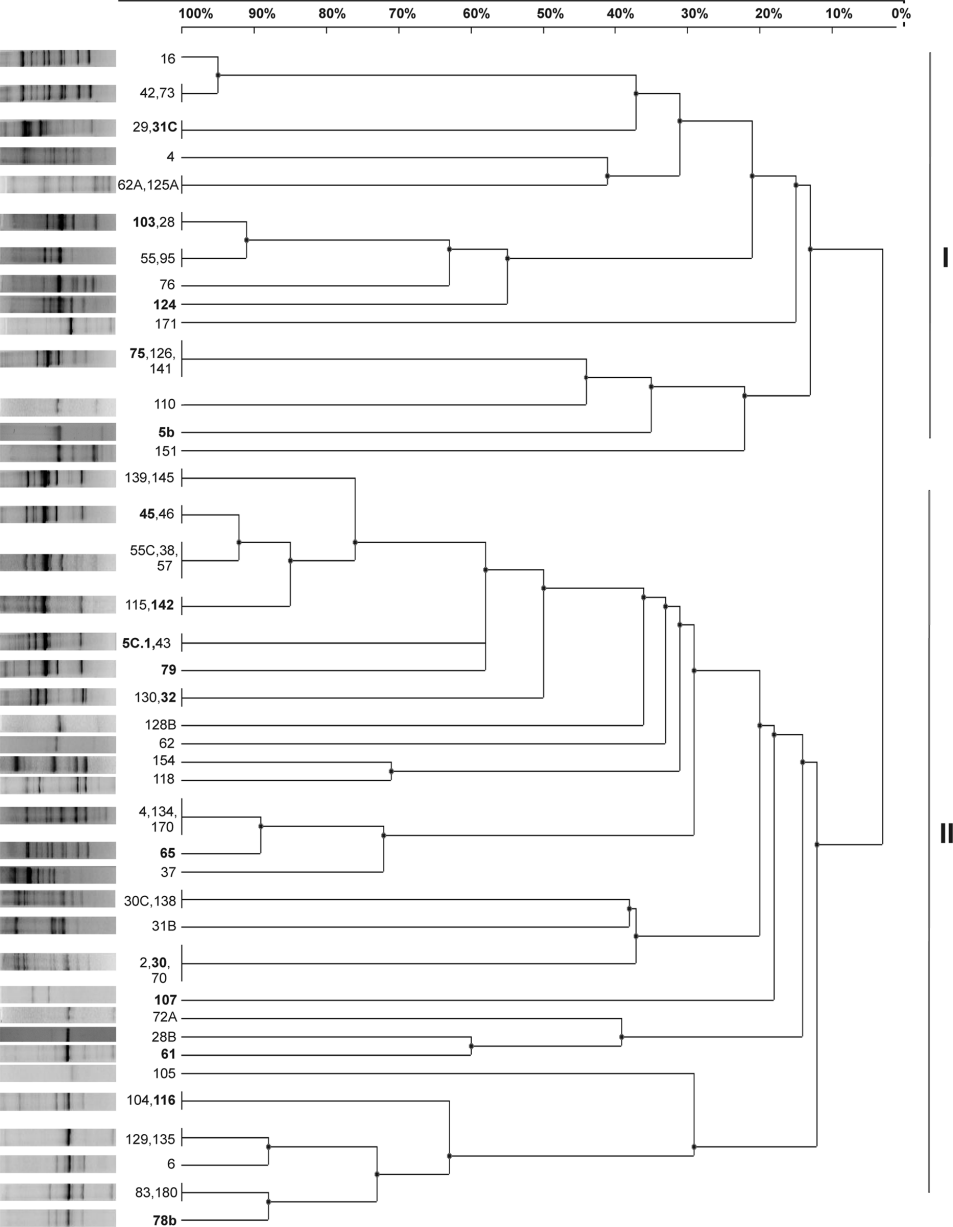
The dendrogram constructed based on BOX-PCR fingerprint profiles (on the *left*) of genomic DNA of the 63 *T. rubens* isolates using UPGMA method. The *numbers* represent the different isolates

To examine the genomic diversity of *T. rubens* nodule isolates, DNA of 63 isolates were analyzed using the BOX-PCR analysis. The dendrogram constructed based on these results showed a high genomic diversity of the isolates, which were divided into two large groups at a low-level similarity (~1%) and further separated into numerous branches (Fig. 1). In total, 41 different DNA profiles with 23 characteristic for single isolates were found.

### Plasmid patterns of the *T. rubens* nodule isolates

**Fig. 2:**
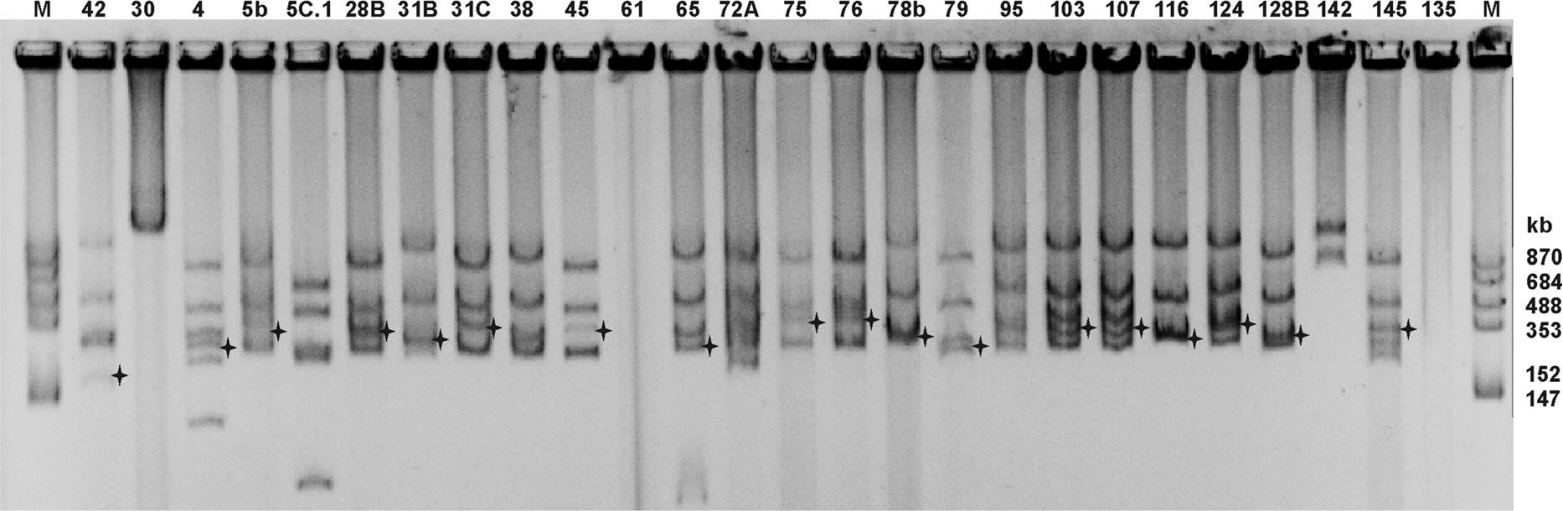
Plasmid profiles of selected *T. rubens* nodule isolates. M—molecular weights of *R. leguminosarum* bv. *viciae* 3841 plasmids, star—symbiotic plasmid

A group of 42 *T. rubens* nodule isolates representing different BOX-PCR profiles was selected and further investigated for the plasmid content using the Eckhardt lysis procedure (Fig. 2). In four isolates, plasmids were not identified. Other strains harbored from 1 to 6 plasmids with approximate molecular weight (m.w.) ranging from 70 to 1140 kb (data not shown). Isolates that differed in the plasmid patterns were assumed to be distinct strains. In a majority of the strains, a single symbiotic plasmid (pSym) with an approximate m.w. ranging from 263 to 458 kb was identified by Southern hybridization with the *nodC* probe derived from *R. leguminosarum* bv. *trifolii* TA1 (Mazur et al. 2011). pSym plasmids were not identified in 10 of the 42 strains. The total m.w. of the plasmids in the individual strains ranged from 1124 to 2885 kb with the average of 2176 kb, which constitutes 30.2% of the average genome size of *R. leguminosarum* bv. *trifolii* WSM2304 and WSM1325 of approx. 7.2 Mb (Reeve et al. 2010a, b). Table 1 shows the main symbiotic and genetic differences between the examined *T. rubens* isolates.

**Table 1:** Genetic and symbiotic differences among *Trifolium rubens* nodule isolates

Strain	Nodulation of clover	Number of plasmids	BOX group
Trb5C.1	+	5	II
Trb45	+	4	II
Trb65	+	5	II
Trb75	+	4	I
Trb78b	+	3	II
Trb107	+	5	II
Trb116	+	3	II
Trb124	+	4	I
Trb30	–	1	II
Trb32	–	4	II
Trb61	–	–	II
Trb142	–	2	II

### Phylogenetic analysis of *T. rubens* isolates

**Fig. 3:**
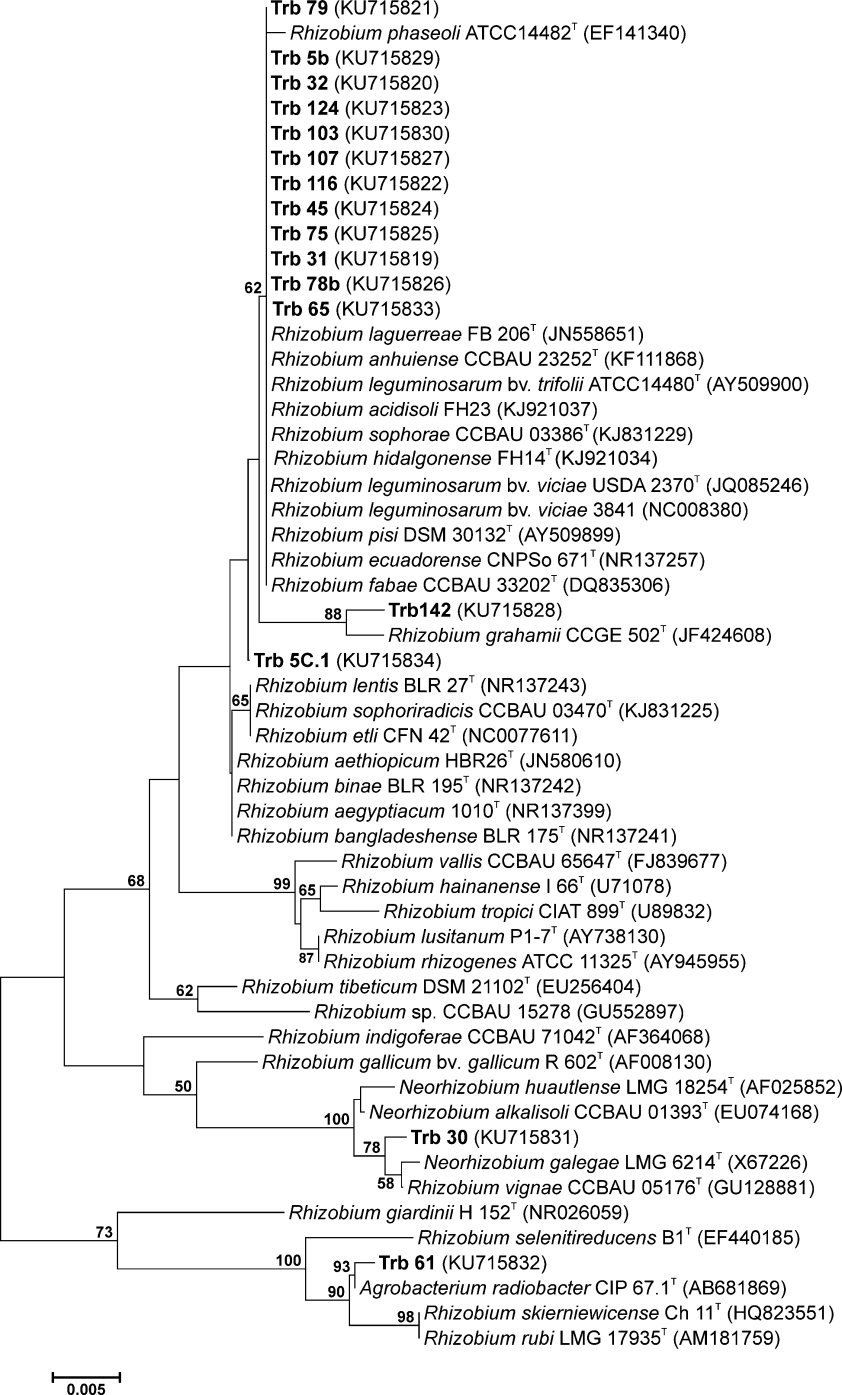
Neighbor-joining (NJ) phylogenetic tree inferred based on partial (786 bp) of 16S rRNA gene sequences of *T. rubens* isolates. The GenBank accession number for each strain is shown in parentheses. The *scale bar* indicates the number of nucleotide substitutions per 100 nt

**Fig. 4:**
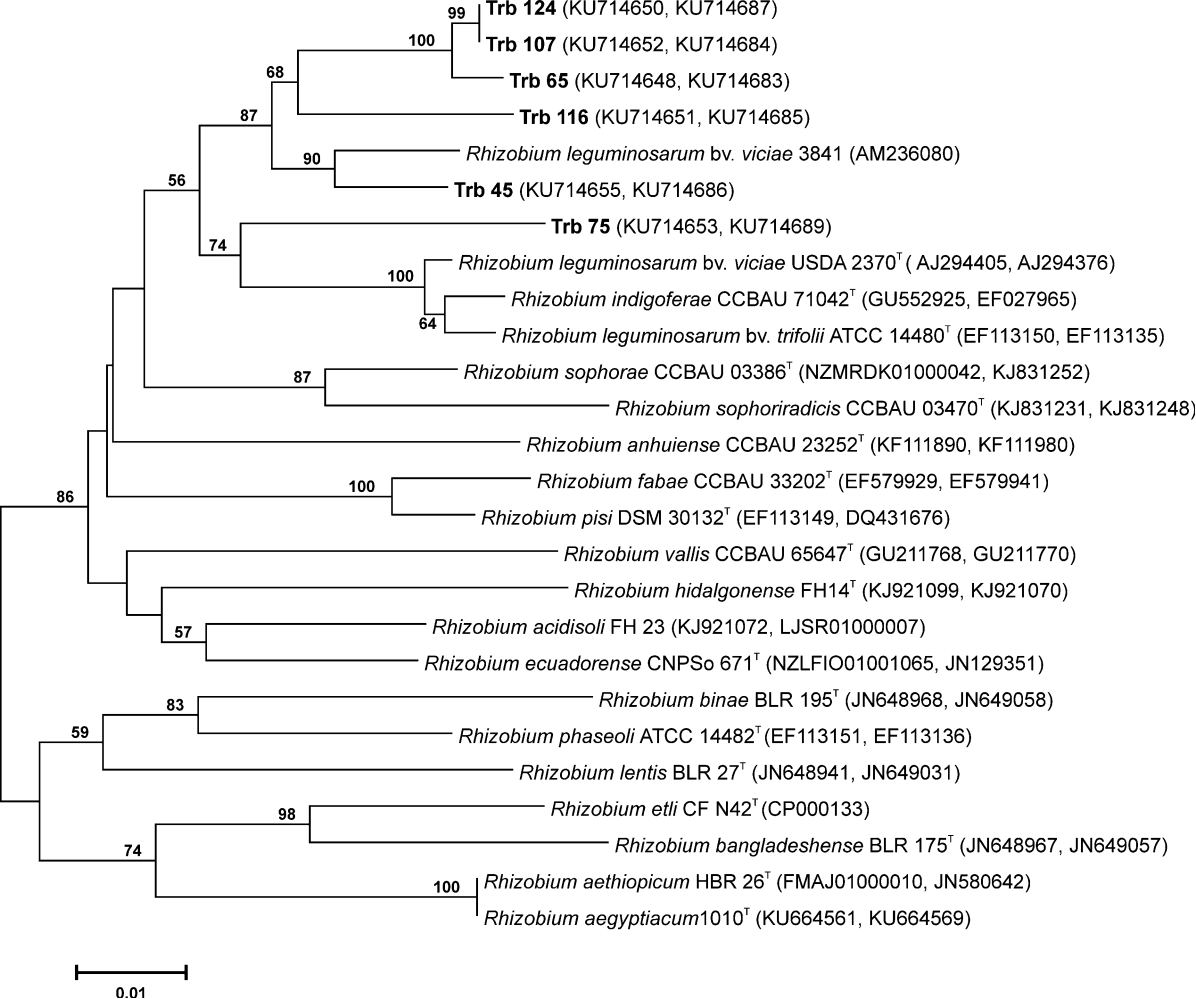
Neighbor-joining (NJ) phylogenetic tree inferred based on concatenated sequences (759 bp) of *atpD* and *recA* genes of *T. rubens* isolates. The GenBank accession number for each strain is shown in parentheses. The *scale bar* indicates the number of nucleotide substitutions per 100 nt

To determine the taxonomic status of *T. rubens* symbionts, fragments of 16S rDNA of 16 isolates were sequenced for genus identification. Phylogenetic analysis showed unambiguously a close relationship of these isolates to the type strains of *Rhizobium* spp. with 99.5–100% 16S rDNA sequence similarity (Fig. 3). Twelve of the 16 strains formed a separate, monophyletic group together with the *Rhizobium* spp. type strains with 100% similarity. Three strains were clearly distinct from the other isolates: strain Trb61 was closely related to *Agrobacterium radiobacter* CIP67.1^T^ with 100% similarity and strain Trb30 was related to *Neorhizobium galegae* LMG6214^T^ and *R. vignae* CCBAU05176^T^ with pairwise sequence similarity 99.6 and 99.7%, respectively. Strain Trb142 was most similar (99.4%) to *R. grahamii* CCGE502^T^. For further species identification, the *atpD* and *recA* chromosomal genes of *T. rubens* isolates were sequenced. The phylogenetic trees constructed for the individual core genes using the neighbor-joining method (NJ) with high bootstrap replications showed that the strains which nodulate *T. repens* belong to the *R. leguminosarum* species (data not shown). Next, the concatenated sequences (759 bp) of two core genes (*atpD, recA*) were subjected to the NJ analyses, which allowed species identification of the isolates (Fig. 4). The topology of the phylogenetic tree was found to be very similar to that of the individual gene trees. The 6 isolates (Trb124, 107, 65, 116, 45, 75) forming the separate cluster were the most similar to *R. leguminosarum* bv*. viciae* 3841 (Young et al. 2006) (97.8–95.4%), *R. leguminosarum* bv*. viciae* USDA2370 (95.6–94.9%), *R. leguminosarum* bv. *trifolii* WSM1325 (Reeve et al. 2010b) and *R. indigoferae* CCBA71042^T^ (95.1–94.3%) (Wu et al. 2011) showing their common taxonomic position (Table 2). The pairwise comparisons of the *T. rubens* isolates to the other relatives were 94.3–93.5% similar to *R. pisi* DSM 30132^T^, 93–94% to *R. fabae* CCBAU33202^T^, and 93–94% to *R. leguminosarum* bv. *trifolii* WSM2304. Strain Trb30 was most closely related to *R. vignae* CCBAU05176^T^ and *N. galegae* LMG6214^T^ with 97 and 96% similarity, respectively (Ren et al. 2011; Österman et al. 2015). *Rhizobium* sp. strain Trb142 was similar to *Rhizobium* sp. CCBAU15278 (92.5%) (Wu et al. 2011), but its taxonomic position remains unclear. The most distinct Trb61 was identified as *A. radiobacter* CIP67.1^T^ with 99.5% similarity.

**Table 2:** Sequence similarities (%) of 16S rDNA, concatenated *atpD*, *recA*, and *nodC* between *Trifolium rubens* nodule isolates and the closely related *Rhizobium* spp

Strains	Trb45			Trb65			Trb75			Trb107			Trb116			Trb124		
	16S	*atpD recA*	*nod*C	16S	*atpD recA*	*nod*C	16S	*atpD recA*	*nod*C	16S	*atpD recA*	*nod*C	16S	*atpD recA*	*nod*C	16S	*atpD recA*	*nod*C
*R. leguminosarum* bv. *viciae* 3841	100	97.8	73.6	100	96.6	73.4	100	95.4	74.2	100	96.4	74.2	100	96.1	74.2	100	96.4	74.2
*R.leguminosarum* bv. *viciae* USDA 2370^T^	100	95.6	74.2	100	94.7	73.4	100	95.4	74.8	100	94.9	74.8	100	95.0	74.8	100	94.9	74.8
*R. indigoferae* CCBAU 71042^T^	97.3	95.0	–	97.3	94.2	–	97.3	95.1	–	97.3	94.3	–	97.3	94.4	–	97.3	94.3	–
*R. leguminosarum* bv*. trifolii* ATCC14480^T^	99.8	95.1	96.7	99.8	94.3	93.8	99.8	95.0	96.7	99.8	94.4	96.7	99.8	94.4	96.7	99.8	94.4	96.7
*R. sophorae* CCBAU 03386^T^	100	95.4	67.0	100	95.0	66.8	100	94.2	67.2	100	95.1	67.2	100	95.0	67.2	100	95.1	67.2
*R. sophoriradicis* CCBAU 03470^T^	99.5	93.2	67.0	99.5	92.5	67.0	99.5	91.8	67.2	99.5	92.6	67.2	99.5	92.0	67.2	99.5	92.6	67.2
*R. anhuiense* CCBAU 23252^T^	100	94.2	72.6	100	93.3	72.6	100	92.7	73.2	100	93.5	73.2	100	93.3	73.2	100	93.5	73.2
*R. aegyptiacum* 1010^T^	99.7	91.8	97.3	99.7	90.6	94.2	99.7	91.3	97.3	99.7	91.1	97.3	99.7	91.3	97.3	99.7	91.1	97.3

**Fig. 5:**
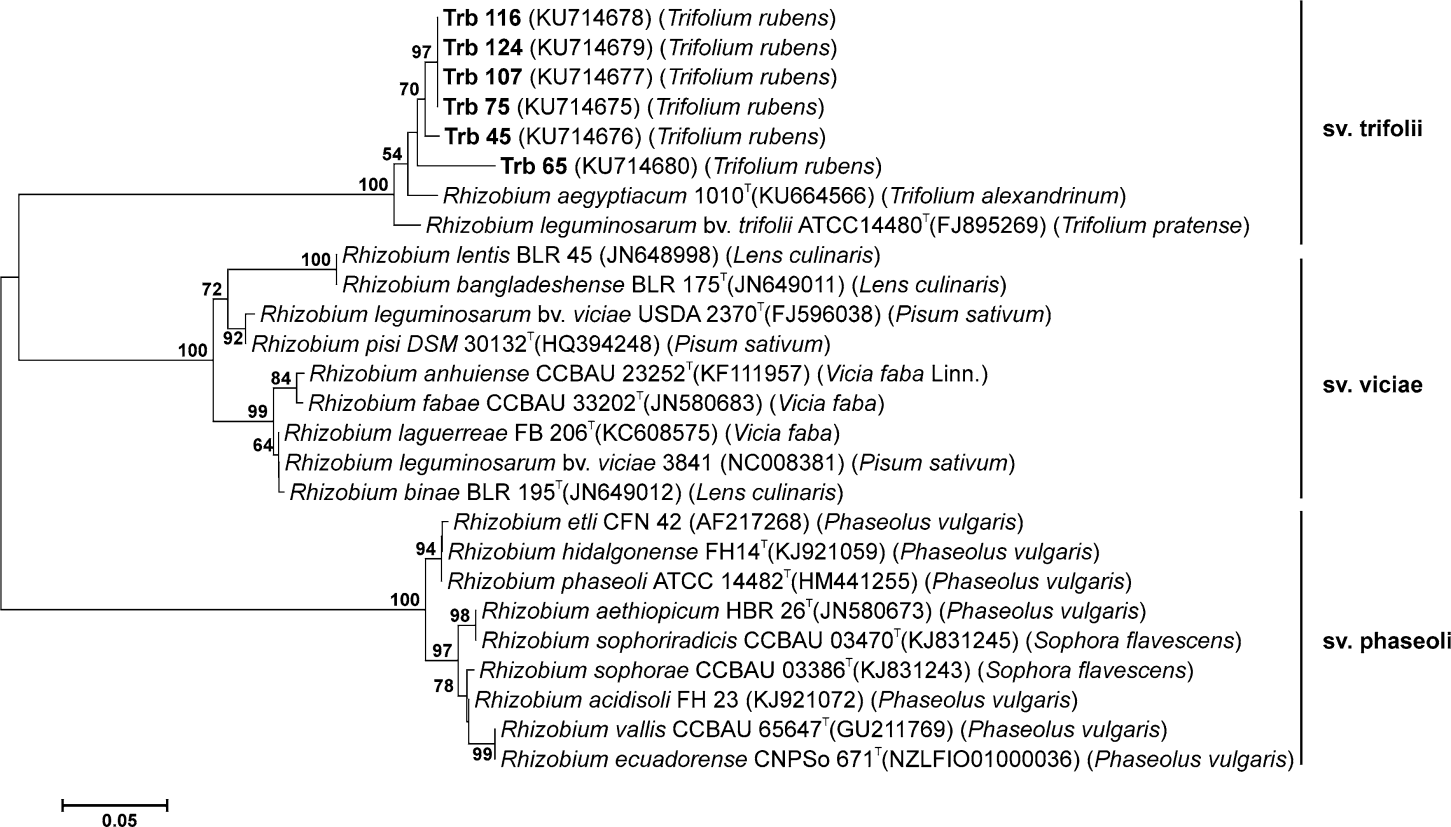
Neighbor-joining (NJ) phylogenetic tree inferred based on *nodC* sequences (372 bp) of *T. rubens* isolates. The GenBank accession number for each strain is shown in parentheses. The *scale bar* indicates the number of nucleotide substitutions per 100 nt

To examine the phylogenic history of symbiotic genes, the common *nodC* gene encoding N-acetylglucosaminyltransferase engaged in the first step of Nod factor synthesis was sequenced in the isolates identified as most similar to *R. leguminosarum* and the NJ phylogenetic tree was constructed (Fig. 5). The sampled *T. rubens* isolates formed a separate cluster with 97.3–100% sequence identity to one another. In this cluster, 4 isolates (Trb116, 124, 107, 75) possessed identical *nodC,* but some minor differences in *nodC* were found in strain Trb45, which showed 98.8% identity and in strain Trb65 with 95.8% identity with the other isolates. The *nodC* of 6 strains was highly similar to the *nodC* of *R. leguminosarum* bv. *trifolii* ATCC14480^T^, with sequence identity 96.7 and 93.8% in Trb65 (Table 2). Interestingly, the *nodC* sequences of *T. rubens* isolates were most similar (97.3 and 94.2% in Trb65) to *nodC* of the recently described new species *Rhizobium aegyptiacum* USDA 7124^T^ isolated from nodules of *Trifolium alexandrinum* L., clover plants growing in different regions in Egypt and closely related to *Rhizobium bangladeshense* BLR175^T^ isolated from lentil (Shamseldin et al. 2016; Rashid et al. 2015). Housekeeping chromosomal genes *atpD* and *recA* of *R. aegyptiacum* USDA 7124^T^ were distantly related with 91.8–91.1% sequence similarity to the genes of *T. rubens* isolates, that confirms their belonging to different species and the same symbiovar *trifolii*. Other *Rhizobium* spp. strains form clearly separated symbiovars *viciae* and *phaseoli* (Fig. 5).

Based on the analysis of symbiotic phylogeny, 6 of the 9 sampled *R. leguminosarum* strains isolated from *T. rubens* nodules were unambiguously classified into biovar *trifolii.* We did not obtain *nodC* amplicons in the case of strains Trb30, 61, and 142, which is consistent with the negative result of Southern hybridization with the *nodC* probe in the plasmids assay (Fig. 2). Since these isolates were classified into the *Rhizobium* genus (Fig. 3), a significant polymorphism in *nodC* alleles or lack of *nodC* (e.g. Trb61) might be the causes of the lack of the *nodC* amplicons in these strains.

### Host specificity and symbiotic efficiency of *T. rubens* nodule isolates

The selected *T. rubens* nodule isolates assigned to the genus *Rhizobium* (Fig. 3) were used for inoculation of clover plants in the tube experiments. Besides the red feather clover (*T. rubens),* the most commonly cultivated clovers were used: red clover (*T. pratense* L. cv. Rozeta), white clover (*T. repens* cv. Lipollo), and Persian clover (*T. resupinatum* L. Lightning). In the laboratory experiment, the clover plants were inoculated with 12 isolates and grown for 5 weeks. Only 8 strains classified as *R. leguminosarum* bv. *trifolii* (Table S2) formed nodules on the clovers. *T. rubens* nodulated by these strains formed 2.7–4.6 nodules per plant; the nodules were mostly small and white. The efficiency of nodulation measured as fresh weight of aerial clover parts was low, ranging from a 0.9 to 1.2-fold value (average 1.04) of fresh weight of shoots in relation to the uninoculated control. Only in the case of 3 (Trb5C.1, Trb65, Trb75) of the 8 isolates was the shoot weight difference significant, however, the symbiotic productivity was very low (Table S2). The other clovers i.e. *T. pratense, T. repens*, and *T. resupinatum* were nodulated by the same 8 rhizobial strains with various symbiotic effectiveness. In the case of *T. pratense,* 5 of the 8 strains formed low effective symbiosis (shoot weight average 1.8). In *T. repens,* 7 of the 8 strains showed increased fresh shoot weight (1.9 average), but the differences in shoot weight were significant only in 3 strains. The highest symbiotic productivity was recorded in Persian clover (*T. resupinatum*), and symbiotic efficiencies of 6 of the 8 strains were significant (2.6 average) relative to the uninoculated control. Strain Trb75, which inoculated four species of clover with a significant level of nitrogen fixation, was the most effective symbiont. Strains Trb116 and Trb78b induced the highest number of nodules on *T. pratense* and *T. resupinatum*; however, in both cases, the symbioses were inefficient and the values of the plant shoot weight were below those of the control plants.

In the pot plant assay, the most efficient *T. rubens* isolates (Trb45, Trb65, Trb75, Trb124) were selected for inoculation of four *Trifolium* spp. (Table 3; Fig. S1). The results confirmed the very low symbiotic efficiency of *Rlt* isolates in symbiosis with their native host *T. rubens*. Only in the case of Trb75 was the wet mass of aerial parts of clover over twice as much as that of the control. As in the tube assay, the most productive was the symbiosis of *T. resupinatum* with all the studied strains, yielding significant differences in wet aerial mass in all cases and from 3.9- to 13.6-fold higher wet mass. The symbiotic productivity of *T. pratense* was low and similar in four *Rlt* strains, yielding from 1.4- to 2.3-fold higher green plant mass. In the *T. repens* –*Rlt* association, the increase in the wet mass of plants inoculated with the studied strains was significant relative to control (fold 2.2–3.7); however, it was still lower than the *T. resupinatum* symbiotic productivity (Fig. S1). In conclusion, the efficiency of symbiosis of *T. rubens* isolates belonging to *Rlt* is host plant- and strain-dependent, indicating in most cases lack of symbiotic compatibility between rhizobia and *T. rubens, T. pratense* and T. *repens* host plants. The most compatible host plant for *T. rubens* isolates appeared to be *T. resupinatum* (Persian clover).

**Table 3:** Host range and symbiotic efficiencies of *Trifolium rubens* nodule isolates—plant pot experiment

Strains	*T. rubens* L.			*T. pratense* var. Rozeta			*T. repens* var. Lipollo			*T. repens* var. Lipollo		
	Nodule no./plant	Fresh weight of shoots (mg)	Fold of shoot weight	Nodule no./plant	Fresh weight of shoots (mg)	Fold of shoot weight	Nodule no./plant	Fresh weight of shoots (mg)	Fold of shoot weight	Nodule no./plant	Fresh weight of shoots (mg)	Fold of shoot weight
Trb45	22.4 ± 10.2	20.8 ± 9.6	0.9	22 ± 6.2	160.1 ± 53.9*	2.1	5.7 ± 1.7	136.5 ± 26.7*	3.7	17.1 ± 4.3	183.8 ± 83.1*	3.9
Trb65	8.5 ± 1.9	23.1 ± 7.8	1	14.1 ± 2.9	106.3 ± 54.6	1.4	8.5 ± 2.7	81.6 ± 29.0*	2.2	22.2 ± 3.6	180.9 ± 43.9*	3.8
Trb75	14 ± 2.6	50.8 ± 17.0*	2.2	10.0 ± 5	177.8 ± 87.9*	2.3	5.2 ± 1.8	111.6 ± 29.1*	3	14.7 ± 4.8	643.6 ± 206.8*	13.6
Trb124	10.3 ± 4.4	36.9 ± 20.0	1.6	19.1 ± 4.3	170.9 ± 51.3*	2.2	4 ± 1.6	119.4 ± 40.7*	3.3	17.8 ± 5.4	369.6 ± 186.3*	7.8
Control un-inoculated (N+)	0	28.5 ± 11.1	1.2	0	133.1 ± 23.4	1.7	0	51.2 ± 13.1	1.4	0	223.3 ± 86.3	4.7
Control un-inoculated (N−)	0	23.1 ± 10.8	1.0	0	76.2 ± 28.5	1.0	0	36.6 ± 12.1	1.0	0	47.3 ± 16.4	1.0

Data were analyzed using Student’s test

* significant difference P ≤ 0.05

To study nodule organization, the 5-week-old nodules of *T. rubens* induced by effective (Trb75), low effective (Trb124), and ineffective (Trb65) strains were observed in confocal microscopy using a live/dead staining procedure involving application of a mixture of nucleic acid fluorescent dyes, SYTO09 and PI, to clover nodule sections (Fig. S2a, S2b). In the case of efficient nitrogen fixing nodules infected by Trb75, distinct developmental zones, i.e., a meristem zone (I), infection zone (II), and nitrogen fixation zone (III) were seen (Fig. S2b). The green fluorescent rhizobia filled the whole nitrogen-fixation zone, indicating that the bacteroids were live and metabolically active. However, in the same zone, numerous, red fluorescent dead bacteria were also observed (Fig. S2a). In the nodules infected by low effective Trb124, green live cells were clearly seen in the infection zone (II) and a few, green fluorescent live cells occupied the root-proximal oldest zone (IV); in the nitrogen-fixation zone, no live rhizobia were observed showing early abortion of bacteroid development. In the case of Trb65, which inefficiently infected *T. rubens,* only the infection zone with red fluorescent bacteria was seen; dead bacteria occupied the whole nodule indicating that the bacteria were rapidly killed and nodule development was arrested at the first stage of plant cell infection.

Besides the specific rhizobia infecting *T. rubens,* isolates that did not nodulate the clover species were found (Table S2). Among these, strain Trb30 was classified as most similar to *R. galegae* and *R. vignae,* strain Trb32 to *R. phaseoli* (data not shown), and Trb142 as *Rhizobium* sp. CCBAU15278 was most similar (89.6%) to *R. grahamii* CCGE502. Strain Trb61.2 was classified as *A. radiobacter* CIP67.1^T^ (Fig. 3). Only in the case of Trb142-inoculated *T. repens* was some increase in plant growth observed (on average 1.6-fold, relative to the control) showing some plant promoting activity (Table S2).

## Discussion

The present study revealed variable symbiotic responses of the host *T. rubens*, *T. pratense, T. repens*, and *T. resupinatum* clover species to inoculation with *R. leguminosarum* bv. *trifolii* isolated from *T. rubens* nodules. Both in the tube and pot plant assays, a majority of *Rlt* isolates from T. *rubens* nodules developed inefficient symbioses with their native host or weakly efficient with association with *T. pratense* and *T. repens*. The most productive, compatible symbiosis of *T. rubens* isolates was observed in the case of the agronomical important species *T. resupinatum* (Persian clover).

Several active *nifHDKEN*, *nifB* and *fixA* genes are required for Fix^+^ phenotype of *Rlt* (Perret et al. 2000). Commonly, *Rlt* strains isolated from red, white, or Persian clovers nodulated efficiently their original host plants (Beauregard et al. 2004). However, various responses of host plants from the same cross-inoculation group or the same species to a particular rhizobial strain have also been described, but the cause of this symbiotic incompatibility was mostly unexplained (Balatti and Pueppke 1992; Tesfaye and Holl 1999; Beauregard et al. 2004; Brito et al. 2008; Melino et al. 2012). Miller et al. (2007) described efficient symbiosis of the *Rlt* strain with *T. ambiguum* (Caucasian clover) and inefficient symbiosis with *T. repens.* In a molecular study of the *nif/fix* genes of this symbiont, the activity of the *nifH*-*fixA* intergenic region binding regulatory proteins was shown to be necessary for *nifH* expression which was responsible for host-specific efficient symbiosis of *Rlt* with *T. ambiguum.* Changes in the nucleotide sequence in the intergenic region affected the *nifH* expression resulting in the Fix^−^ symbiotic phenotype (Miller et al. 2007). Other reports also suggested several regulatory mechanisms involved in host-specific symbiosis such as interaction of RpoN with the *nifH* promoter (Michiels et al. 1998). Cebolla et al. (1994) found that expression of *Ensifer meliloti nifH* and *fixA* promoters was very low in heterologous rhizobial backgrounds in comparison to homologous ones, which may be essential for host specificity. This effect was most strongly seen in the *Rlt* background. These observations show that optimal symbiotic nitrogen-fixation depends on mostly unknown plant and microbial regulatory factors.

Recently, a specific role of the NCR (NCR169) peptide in the differentiation and persistence of nitrogen fixing bacteroids in *Medicago truncatula* has been documented (Horváth et al. 2015; Price et al. 2015). Lack of this peptide in a *M. truncatula* mutant caused ineffective symbiosis with the specific symbiont. In the plant mutant, the bacterial differentiation was impaired and early senescence of symbiotic cells occurred. Complementation of mutation was associated with restoration of N_2_ fixation (Horváth et al. 2015). Price et al. (2015) found that the strain-host dependent symbiotic inefficiency in *M. truncatula*—*E. meliloti* interaction was dependent on specific degradation of NCR169 by a bacterial enzyme, zinc-metallopeptidase, encoded by gene *hrrP* located on the accessory plasmid. Lack of NCR169 affected the symbiotic outcome in late nodule development by triggering premature degeneration of differentiated nitrogen-fixing bacteroids and caused nodule senescence. The plasmid elimination or mutation in the *hrrP* gene caused the Fix^+^ phenotype of *E. meliloti* (Price et al. 2015). A critical role for symbiosis is also played by the BacA protein, which protects *E. meliloti* against the anti-bacterial action of NCR peptides allowing bacterial persistence within the nodule (Haag et al. 2011).

Since the symbionts of *T. rubens* have not been genetically described so far, the analyses of the genomic diversity and symbiotic phylogeny of the nodule isolates were performed. The pangenome of *Rlt* (Medini et al. 2005), consists of several strains with core genes shared by all the strains and a set of genes influencing the phenotypes and symbiotic activity of the strains (Kumar et al. 2015). A characteristic feature of the species is that it harbors several plasmids comprising a considerable number of accessory genes, which supports the broad genetic and phenotypic diversity of strains and species. The plasmids play a crucial role in rhizobial adaptation to specific environmental niches and strain evolution (Palacios and Newton 2005; Mazur and Koper 2012). The symbiotic genes of *R. leguminosarum* located on the pSym plasmid were also found to be highly variable and affected efficient symbioses with several host plants (Ramirez-Bahena et al. 2008, 2009; Marek-Kozaczuk et al. 2013). In this study, the genomes of the *T. rubens* nodule isolates were found to be highly differentiated based on the plasmid content showing considerable genetic variability as described earlier for *Rlt* isolated from nodules of *T. pratense* (Mazur et al. 2011, 2013). The taxonomic position of the selected *T. rubens* isolates was unambiguously defined as *R. leguminosarum*. The symbiotic phylogeny based of the *nodC* genes allowed defining the symbiovar of 6 isolates as *Rlt* that nodulated *T. rubens, T. pratense, T. repens*, and *T. resupinatum*. Fluorescence microscopy of Fix^+^ *T. rubens* nodule sections (Trb75) showed proper zonation; several plant cells were occupied by metabolically active bacteroids showing correct nodule development. The Fix^+^/Fix^−^ *T. rubens* nodule section (Trb124) showed that the development of rhizobial infection was arrested in the infection zone and only a few plant cells in the oldest zone contained viable bacteroids reflecting residual nitrogen fixation observed in plant tests. In the Fix^−^ *T. rubens* nodule sections (Trb65), the infection was also aborted in the infection zone but a vast majority of rhizobia were dead, corroborating inefficient symbiosis. Altogether, the morphological changes in nodule development confirmed the variable symbiotic activity in the interaction of the *Rlt* strains with the native clover *T. rubens*. We can speculate that some bacterial or plant molecular factors taking part in the later stages of nodule development, such as bacteroid terminal differentiation to nitrogen fixing forms are responsible for the different responses of the host plant to specific symbionts. These results show that the specificity in the clover symbiosis may not be limited to nodule formation, but might also be controlled at later stages of nodule development (Tesfaye and Holl 1999; Wielbo et al. 2010a; Price et al. 2015). Currently, the role of NCR plant peptides in the *Trifolium* - *R. leguminosarum* interactions has not been explored, but it is tempting to consider the involvement of the plasmid located genes in affecting the symbiotic phenotype of some host-rhizobia associations, especially in *Rlt* strains harboring several accessory plasmids. The mechanism by which a majority of *Rlt* isolates from *T. rubens* nodules do not fix nitrogen in symbiosis with their native *T. rubens* host is currently not understood.

The specific compatible rhizobia play the main role in plant growth, which is a consequence of nitrogen fixation. The legume root nodules are a specific environmental niche induced by competent rhizobia; however, multiple parasitic, symbiotic or endophytic species of bacteria can co-exist inside nodules despite the high selectivity of legume plants towards microsymbionts (Saidi et al. 2014; Sessitch et al. 2012). Endophytes are able to colonize plant internal tissues with minimal or no host damage (Schumpp and Deakin 2010; Zgadzaj et al. 2015). In this work, besides the *Rlt* strains isolated from the *T. rubens* nodules, we found strains that were highly similar to other species such as Trb61—*A. radiobacter* CIP67.1, Trb30—*R. vignae*, Trb142—*R. grahamii* CCGE 502, and several others with an undetermined taxonomic status. These rhizobia can populate nodules due to the reduced selectivity of *T. rubens* host plants towards symbionts. Poorly efficient symbiosis with *T. rubens* can favor non-specific infection, and symbionts and other bacteria coexisting in the nodules form some kind of facultative mutualism. These results are consistent with the hypothesis that the specificity in the clover symbiosis may not be limited to nodule establishment but might also be reflected at later stages of nodulation and/or development of effective nitrogen fixation (Schumpp and Deakin 2010).

## Electronic supplementary material

Below is the link to the electronic supplementary material.

Fig. S1 Symbiotic performance (fresh soot weight and number of nodules per plant) of rhizobial strains Trb45, Trb65, Trb75, Trb124 with (**a**) *Trifolium rubens*, (**b**) *T. pretense* var. Rozeta, (**c**) *T. repens* var. Lipollo, (**d**) *T. resupinatum* var. Ligthning. +N uninoculated plant in medium supplemented with N source. −N negative control (uninoculated plant). Average values with standard deviations are shown. *Asterisks* indicate statistical significant differences (P value < 0.05). Supplementary material 1 (TIFF 511 kb)

Fig. S2a. Confocal microscopy of nodule sections of *Trifolium rubens* inoculated with *R. leguminosarum* bv. *trifolii* Trb65, Trb75, Trb124 isolates presenting different symbiotic clover response. Nodules were stained with a mixture of SYTO9 (*green signal*) and PI (*red*). Live bacteria are stained by SYTO9 and dead bacteria with PI; co-localization *green* and *red* (merge). Supplementary material 2 (TIFF 412 kb)

Fig. S2b. Confocal microscopy of nodule section of *Trifolium rubens* inoculated with *R. leguminosarum* bv. *trifolii* Trb75. On the *left*, developmental zones of nodule (I–IV) were marked. Supplementary material 3 (TIFF 790 kb)

Supplementary material 4 (PDF 144 kb)
