# Supplementary figures and images for: Host-dependent symbiotic efficiency of Rhizobium leguminosarum bv. trifolii strains isolated from nodules of Trifolium rubens
Source: Antonie Van Leeuwenhoek. 2017 Aug 8;110(12):1729–44. doi: 10.1007/s10482-017-0922-7 (PMC5676844; doi:10.1007/s10482-017-0922-7)

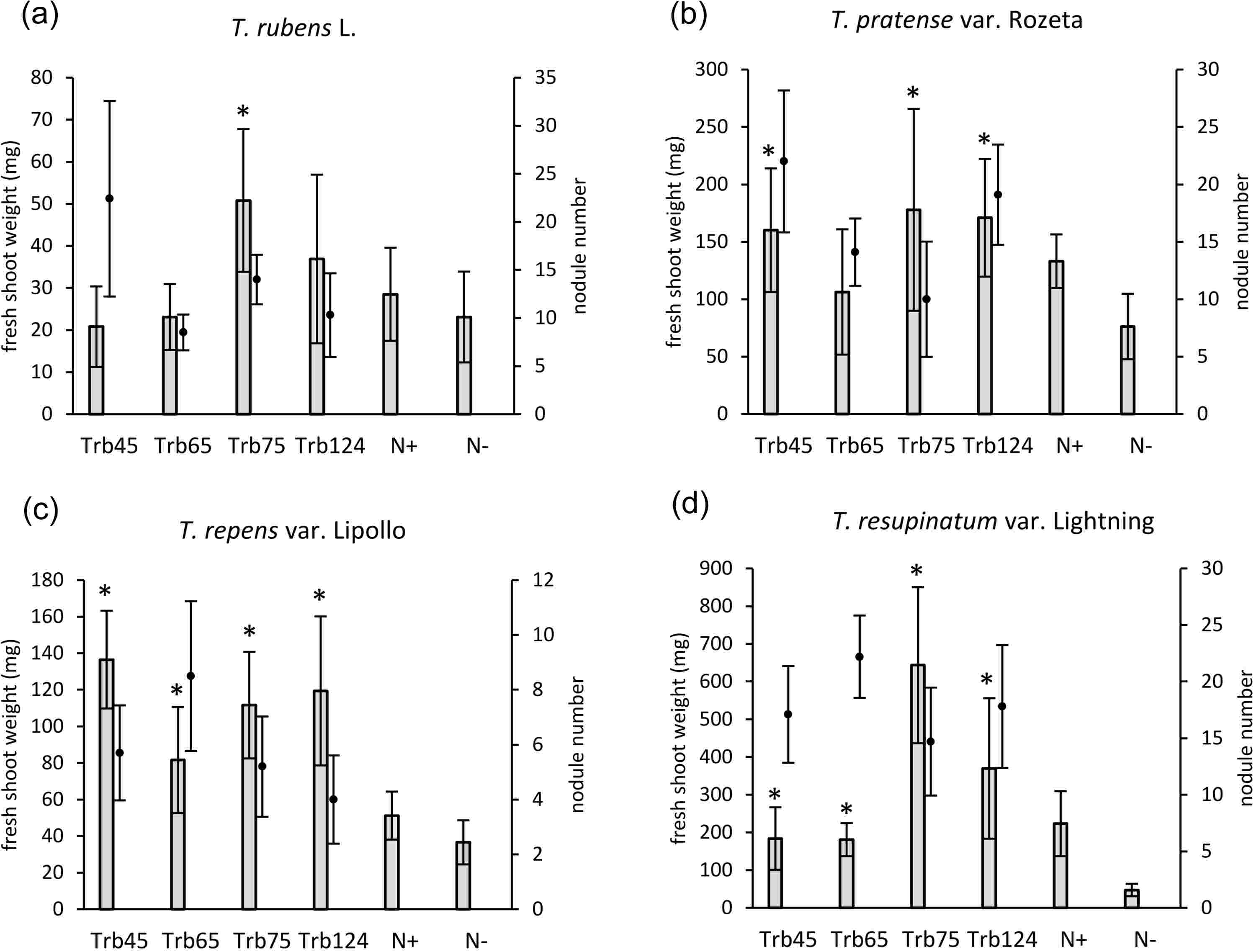

Supplement: Supplementary file 1 — Fig. S1 Symbiotic performance (fresh soot weight and number of nodules per plant) of rhizobial strains Trb45, Trb65, Trb75, Trb124 with (a) Trifolium rubens, (b) T. pretense var. Rozeta, (c) T. repens var. Lipollo, (d) T. resupinatum var. Ligthning. +N uninoculated plant in medium supplemented with N source. −N negative control (uninoculated plant). Average values with standard deviations are shown. Asterisks indicate statistical significant differences (P value < 0.05). Supplementary material 1 (TIFF 511 kb) [file 10482_2017_922_MOESM1_ESM.tif]

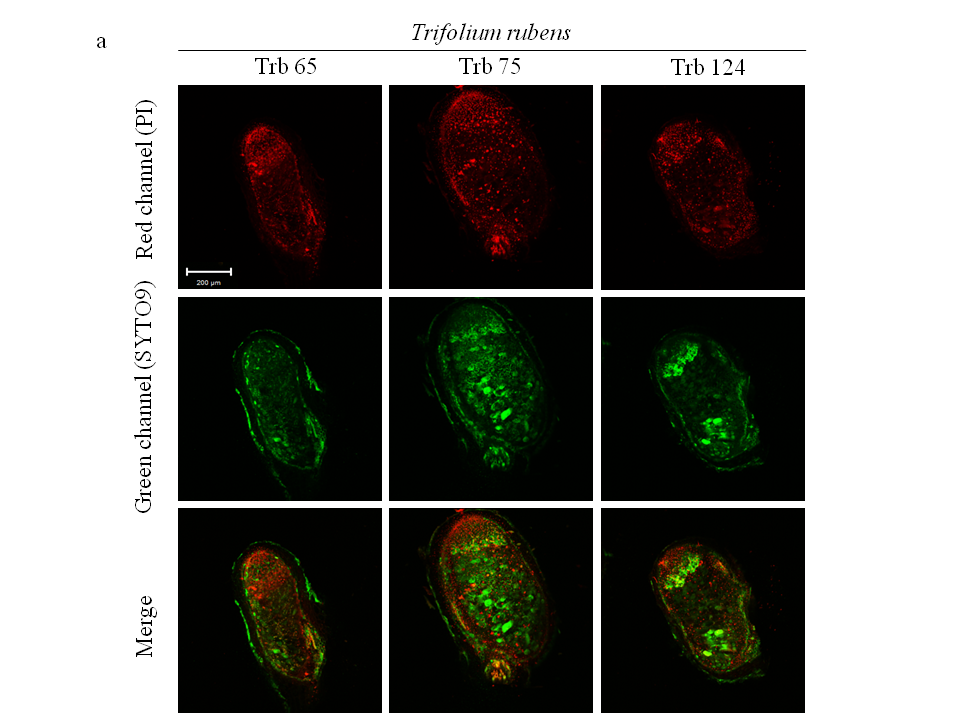

Supplement: Supplementary file 2 — Fig. S2a. Confocal microscopy of nodule sections of Trifolium rubens inoculated with R. leguminosarum bv. trifolii Trb65, Trb75, Trb124 isolates presenting different symbiotic clover response. Nodules were stained with a mixture of SYTO9 (green signal) and PI (red). Live bacteria are stained by SYTO9 and dead bacteria with PI; co-localization green and red (merge). Supplementary material 2 (TIFF 412 kb) [file 10482_2017_922_MOESM2_ESM.tif]

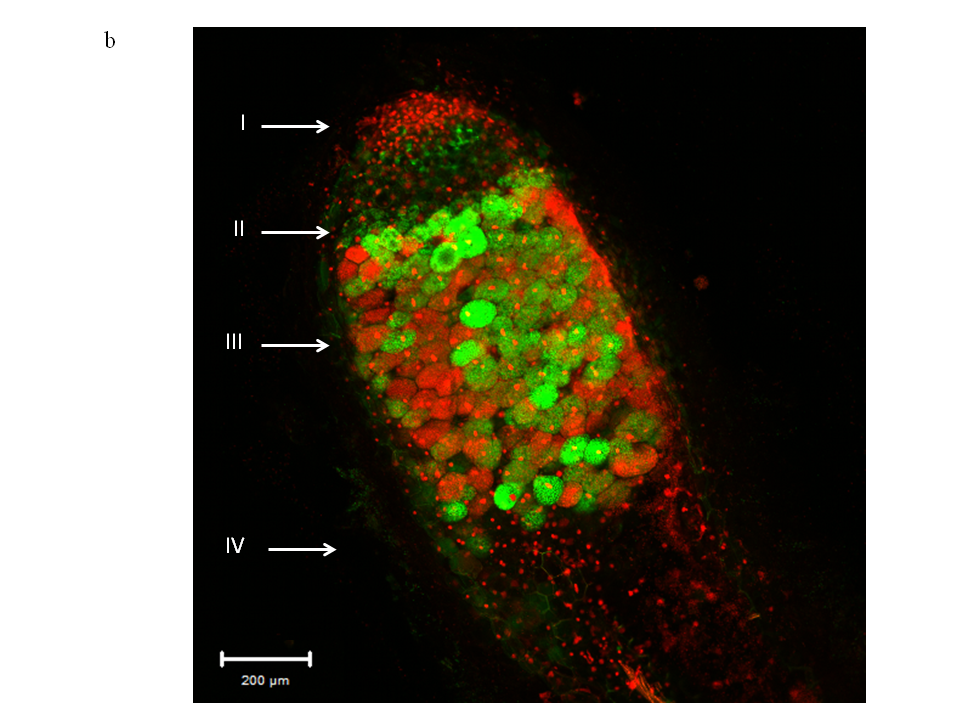

Supplement: Supplementary file 3 — Fig. S2b. Confocal microscopy of nodule section of Trifolium rubens inoculated with R. leguminosarum bv. trifolii Trb75. On the left, developmental zones of nodule (I–IV) were marked. Supplementary material 3 (TIFF 790 kb) [file 10482_2017_922_MOESM3_ESM.tif]
