# Supplementary material for: Host-dependent symbiotic efficiency of Rhizobium leguminosarum bv. trifolii strains isolated from nodules of Trifolium rubens
Source: Antonie Van Leeuwenhoek. 2017 Aug 8;110(12):1729–44. doi: 10.1007/s10482-017-0922-7 (PMC5676844; doi:10.1007/s10482-017-0922-7)
Supplement: Supplementary file 4 — Supplementary material 4 (PDF 144 kb) [file 10482_2017_922_MOESM4_ESM.pdf]

Table S1

Primer	Sequence 5’–3’	Region	Reference
fD1	AGAGTTTGATCCTGGCTCAG	16S rDNA	Weisburg et al.
rD1	AAGGAGGTGATCCAGCC		1991
atpDf1	ATCGGCGAGCCGGTCGACGA	*atpD*	Gaunt et al.
atpDr	GCCGACACTTCCGAACCNGCCTG		2001
recA 6F	CGKCTSGTAGAGGAYAAATCGGTGGA	*recA*	Martens et al.
recA 555R	CGRATCTGGTTGATGAAGATCACCAT		2008
nodCFu	AYGTHGTYGAYGACGGITC	*nodC*	Laguerre et al.
nodCI	CGYGACAGCCANTCKCTATTG		2001
BOX 1AR	CTCCGGCAAGGCGACGCTGAC		Louws et al.
			1994

Table S2

Strains	*T. rubens* L.			*T. pratense* var. Rozeta			*T. repens* var. Lipollo			*T. resupinatum* L. var. Lightning		
	Nodule no./ plant	Fresh weight of shoots (mg)	Fold of shoot weight	Nodule no./ plant	Fresh weight of shoot (mg)	Fold of shoot weight	Nodule no./ plant	Fresh weight of shoots (mg)	Fold of shoot weight	Nodule no./ plant	Fresh weight of shoots (mg)	Fold of shoot weight
Trb5C.1	3.4	36 ±5.9*	1.1	4.5	61.8±13*	2. 2	1.3	22.9±7.4*	2.0	2.9	99±13.8*	3.6
Trb45	4.6	30.1±6.3	0.9	7.6	55±14.7*	1.9	3	17.8±5.7	1.6	2.9	72.3±17.5*	2.6
Trb65	4	40.7±9.7*	1.2	12.9	25.1±7.2	0.9	4.3	14.7±5.6	1.3	4.2	87.8±18.7*	3.2
Trb75	2.7	40.6±9.8*	1.2	4.9	86.3±15.6*	3.0	3	57±15.7*	5.0	2.1	110±10.5*	4.0
Trb78b	3	31.3±7.7	0.9	19.1	23.3±77	0.8	4.3	13.3±3.1	1.2	16.1	26.3±6.9	0.9
Trb107	4.3	29.9±7.8	0.9	7.1	68.7±11.9*	2.4	2.9	13.8±5.6	1.2	3.8	71±1.1*	2.6
Trb116	4.3	31.7±7.8	0.95	16.2	23.5±49	0.8	6	10.8±3.3	0.9	12.6	24.1±5.6	0.9
Trb124	4	39±13.8	1.2	6.5	63.5±17.4*	2.2	3	21.8±5.8*	1.9	3.7	71.6±14.4*	2.6
Trb30	0	25.9±5.5	0.8	0	24.2±9.8	0.8	0	13.7±3.4	1.2	0	21.5±4.9	0.8
Trb32	0	29.8±5.9	0.9	0	20.1±4.9	0.7	0	8.9±2.8	0.8	0	22±4.8	0.8
Trb61	0	29±5.1	0.9	0	26.5±7.7	0.9	0	12±4.5	1.0	0	18.3±3.9	0.7
Trb142	0	36±7.4	1.1	0	28.1±7.5	0.98	0	17.9±6.2	1.6	0	24.8±6.9	0.9
Control un-inoculated	0	33.3±7.9	1.0	0	28.6±7.9	1.0	0	11.5±3.5	1.0	0	27.7±3.8	1.0
